# Closed-loop electrical block of vagus nerve scales from rodent to porcine cardiac models

**DOI:** 10.1088/1741-2552/add8be

**Published:** 2025-05-27

**Authors:** Shane Bender, David Green, Joseph Hadaya, Sahil Haridas, Christopher Chan, Ronald Challita, Al-Hassan Dajani, Jeffery Ardell, Tina Vrabec

**Affiliations:** 1 Department of Physical Medicine and Rehabilitation, Case Western Reserve University School of Medicine, Cleveland, OH, United States of America; 2 Department of Biomedical Engineering, Case Western Reserve University, Cleveland, OH, United States of America; 3 Department of Physical Medicine and Rehabilitation, MetroHealth Medical Center, Cleveland, OH, United States of America; 4 UCLA Cardiac Arrhythmia Center and Neurocardiology Research Program of Excellence, David Geffen School of Medicine at University of California, Los Angeles, Los Angeles, CA, United States of America; 5 Molecular, Cellular, and Integrative Physiology Program, University of California, Los Angeles, Los Angeles, CA, United States of America

**Keywords:** neuromodulation, electrical nerve block, vagus, cardiac, heart rate, closed-loop control, fuzzy logic

## Abstract

*Objective*. Direct current (DC) electrical block of the vagus nerve has shown the ability to downregulate the parasympathetic input to the heart. Previous investigations used static prescribed values, but the main advantage of electrical nerve block is the ability to modulate the block effect in real time. Here we investigate the potential of real-time, closed loop control of heart rate (HR), and how these control schemes translate across species. *Approach.* In anesthetized rats and pigs, proximal vagus stimulation was applied as a perturbation to simulate overactive vagal activity, causing a decrease in HR. DC nerve block was applied distally to mitigate this perturbation and raise HR. The block amplitudes applied were normalized to a block threshold (BT), or the amount of current to block the nerve completely in 60 s. Two static levels of 10% and 50% BT were compared to a closed-loop controlled current. *Main Results.* In both the rat and the pig models, the closed-loop nerve block was able to control the HR to the desired setpoint (SP). Neither of the static values were able to achieve a reliably consistent level of block, with the controlled trials showing a much tighter spread of HR over time. In the pigs, a higher-gain controller was able to reach the SP more quickly. In the rat, the controller reduced both the injected charge and the time to recovery after block. In the pig, the charge was increased, but near-instant recovery times were retained. A closed-loop system is required for precision control of cardiac output. *Significance.* Both the rat and pig models showed success in closed-loop control of HR. Translating from rat to pig models only required minor changes to the controller, indicating that the system is robust. The ease of this translation effort bodes well for potential future translation to human therapies.

## 1. Introduction

### 1.1. Clinical needs

Cardiovascular disease (CVD) is a widespread and growing condition affecting more than a half billion people globally [1, 2]. CVD is associated with significant mortality and morbidity risk and negatively influences one’s ability to conduct activities of daily living. As chronic cardiovascular disease progresses, changes which were initially adaptive, such as tachycardia mediated through increased sympathetic activity, exceed physiologic compensatory mechanisms and become maladaptive, resulting in increased mortality rates [3]. For example, tachycardia, due to either an increase in sympathetic input to the heart or a decrease in parasympathetic tone, is associated with inferior cardiovascular outcomes. These imbalances in neural inputs to the heart increase the chances of arrythmias and sudden cardiac death [4–11]. Arrythmias such as atrial fibrillation can result from high activity from both the sympathetic and parasympathetic inputs [11].

Current treatment modalities primarily emphasize the curtailing of sympathetic tone through beta blockade [12]. These pharmaceuticals are generally used to address tachycardia but cannot be titrated to respond to constantly changing cardiac demand [12]. Other treatments such as pacemakers address bradycardia and but cannot slow down the heart to address tachycardia [13]. Implanted cardiac defibrillators (ICDs) only respond retroactively (i.e. once arrythmia or tachycardia has already begun) by delivering direct current (DC) to the heart to restore sinus rhythm. These cardiac shocks can also have distressing effects for the patient given their conscious state. While these treatments have been shown to be effective for particular patient populations, they do not address issues in the underlying neurological control of cardiac function. A more proactive approach targeting neural pathways that addresses the underlying systemic issues could provide more responsive control [13].

Neuromodulatory treatments are poised to offer newer, more personalized therapeutic options for managing chronic cardiac disease and improving patient outcomes over traditional methods [13–16]. Current surgical techniques for cardiac treatment include neural endpoint ablation and nerve transection, but these techniques are invasive and permanent [17–19]. Vagus nerve stimulation (VNS) is a proven technique to increase parasympathetic input to the heart and shows promise as treatment for ventricular tachycardia and ventricular fibrillation [7–10]. Electrical nerve block can be utilized to downregulate activity on a nerve [20–35], and vagus nerve block has been shown to effectively modulate heart rate (HR) by partially blocking action potential conduction [13, 36, 37]. By utilizing closed-loop control, the electrical blocking waveform can be modulated to guide the HR towards a setpoint (SP) (target HR) and maintain it [37]. This therapy could be applied to either the vagus nerve (parasympathetic input) as illustrated above, or the sympathetic chain (sympathetic input) [15]. These techniques hold the promise of addressing complex arrhythmia conditions that consist of both bradycardia and tachycardia such as sick sinus syndrome. In the present study, the vagus nerve was initially targeted due to its ease of access for clinical applications. In future experiments, a comparison of this therapy between the vagus and the sympathetic nerve will be explored.

### 1.2. Vagal modulation as a treatment

Previous research applies stimulation to the vagus nerve to increase parasympathetic activity as a treatment to actively prevent arrythmias by counteracting sympathetic activity, as well as inducing remodeling of malfunctioning cardiac innervations [38–40]. However, therapies to *reduce* vagus activity are still in the early stages, with irreversible ablation for chronic atrial fibrillation [17] and vasovagal syncope [18, 19] being the most common mode of parasympathetic nervous input reduction. Electrical nerve block could be a preferable treatment for ablation patients, as ablation is complete, always producing effects, and irreversible, whereas block can provide a gradable and adjustable therapy that can even be turned off as the patient requires. Especially in diseases such as vasovagal syncope, where the vagus can function normally outside of intermittent episodes, normal vagus activity would be preferable, and the downregulation of the vagus can be applied only when an episode is imminent.

### 1.3. DC nerve block as a treatment

In these experiments, the therapy being tested was the application of DC nerve block of the vagus nerve during a synthetic increase in vagal tone elicited from a proximal stimulation electrode (the ‘perturbation’). Blocking the vagus reduces the efferent signals sent to the heart via the vagus, thus lowering parasympathetic drive into the heart. This has the effect of increasing HR, as well as heart contraction force and conduction speed. DC nerve block works by providing a cathodic current to the extracellular space around the nerve, which in turn depolarizes a portion of the axon. By maintaining this current, the nerve cell remains depolarized and stays in an artificial refractory period; this in turn prevents incoming action potentials from being conducted through the blocked region [21]. In the vagus, this has the effect of reducing the parasympathetic signals sent to the heart. The afferent sensory signals of the vagus are also blocked, however, electrodes and waveforms are being developed to selectively modulate portions of the vagus, while leaving other portions unaffected [13, 41]. DC nerve block has temporal properties that effect the block efficacy including an *induction effect*, where block efficacy will improve over time at a constant current, and a *recovery effect* which prolongs the neural conduction block after the application of current has ceased [36, 42–46]. These properties must be considered for accurate implementation of DC nerve block.

### 1.4. Experimental aims

Although varying DC nerve block levels can be chosen to immediately block the nerve and to be instantaneously reversible, these properties depend on the magnitude and duration of the applied block. It has been shown that levels of block below instantaneous block threshold (BT) can produce a delayed complete block (induction effect), while prolonged application of block can result in a delayed recovery to full conduction [36]. These previous studies used static block levels to investigate these effects. In practice, to maximize the effectiveness of electrical neuromodulation and manage the temporal changes in block efficacy, a way to automatically modulate the level and duration of block is required. A closed-loop controller approach was implemented to monitor the HR parameters and respond with an appropriate level of block. The controller chosen was a fuzzy logic controller (FLC), which took in measurements of HR error (ΔHR) and the slope of HR error (ΔHR/Δt) and output a change in block amplitude to reach a target HR SP. This controller was implemented in LabVIEW, with electrocardiogram (ECG) or blood pressure (BP) measurements being the primary measurements made, and with the DC level produced through a current regulated device.

The controller was originally developed and tested in a rat model and was then ported to a pig model. Other than the HR scaling by a factor of 2 (based loosely on the anesthetized HR of each animal), no major changes to the controller were made between the two animal models. With the controller performing well in both models, the FLC has proved to be a very scalable tool. Based on our experience with translation from rat to pig, we expect that the use of FLC would facilitate the translation of these methods from pre-clinical to clinical deployment. *The main goals of these experiments were to demonstrate the efficacy of a closed-loop controller as a method for actively titrating the amplitude of the electrical nerve block to achieve a target HR, and to illustrate that applying DC nerve block at a static level cannot control cardiac output with an acceptable degree of precision*. To do this, tonic vagal tone was synthetically elevated using a proximal VNS electrode to cause the HR to decrease. Electrical nerve block was applied to counteract this perturbation using three different blocking protocols. Nerve block using the closed-loop controller was compared to the effect of two different static block amplitudes, which have been evaluated in previous DC nerve block experiments [36]. This was done to demonstrate the effectiveness of the controller, and to illustrate the potential issues that may occur if the block levels are not actively managed with a controller.

## 2. Methods

### 2.1. Rat surgical preparation

**Figure 1.**
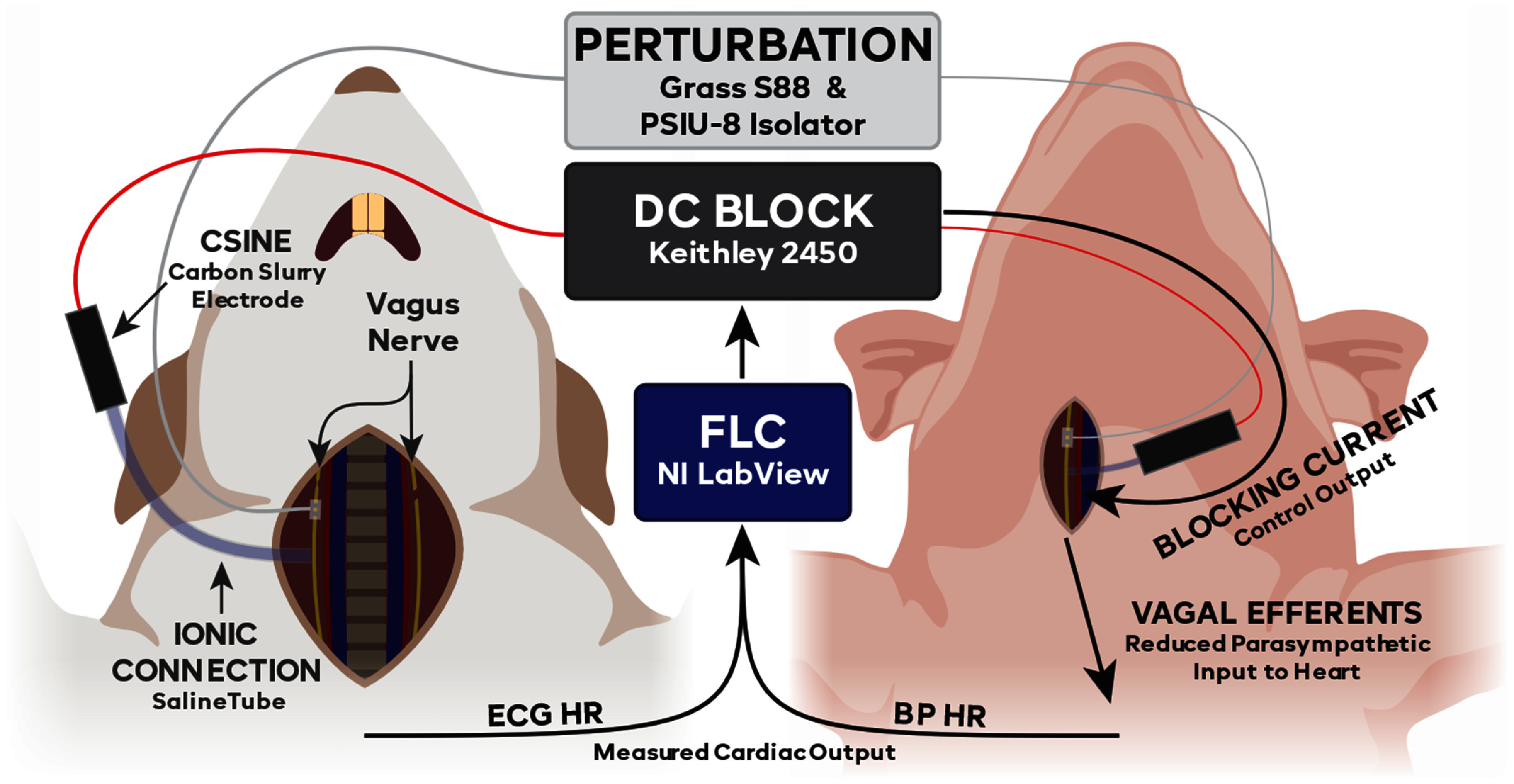
Experimental setup. For both rat and pig experiments, a bipolar stimulating electrode was placed on the proximal right side vagus nerve to deliver the perturbing stimulation, increasing activity on the vagus. The DC current-controlled source was connected to a carbon separated interface nerve electrode (CSINE), which is then ionically connected via a saline bridge to the vagus, distal to the perturbation electrode; this setup allows for the safe delivery of the DC blocking current. For the rats, the right-side nerve was crushed, and the left-side nerve was cut, to remove any basal efferents and any afferent effects caused by the stimulation, which isolated the system to out inputs and increased system stability. In the pig experiments, the vagus nerve on both sides remained intact to more closely model how this therapy would be used in a clinical setting. The fuzzy logic controller (FLC) was fed a measurement signal (electrocardiogram (ECG) or blood pressure (BP)), which was used to calculate the heart rate. The FLC determined what the output would be and directed the current source to output the appropriate blocking current.

Rat experiments were performed under the approval of the Case Western Reserve University (CWRU) Institutional Animal Care and Use Committee (IACUC) under protocol 2014–0151. CWRU is an AAALAC-accredited institution conforming to all applicable federal, state, and local laws and regulations, in addition to institutional policies. Our team was trained and certified by qualified CWRU staff and adheres to Public Health Service policy and the Guide for the Care and Use of Laboratory Animals [47] when working on CWRU IACUC-approved protocols. A total of 6 male Sprague–Dawley rats were used, weighing 400–600 g. The rats were induced using 3%–5% inhaled isoflurane, maintained at 1.5%–3% and intubated with a 14-gauge angiocatheter. Rats were ventilated at 60 breaths per minute with a stroke volume of 4–5 ml. Due to limitations in our setup, this volume was higher than the expected rat tidal volume, but necessary to maintain a deep enough level of anesthesia and proper ventilation. The rat was laid supine, and the cervical portion of the vagus nerve was exposed bilaterally. The left vagus was transected, and the right vagus was crushed at the proximal end of the exposed length. These were done to limit the efferent signals of the vagus to only what was controlled in the experiment, and to remove the afferent effects of the vagal stimulation. This allowed us to test the effects from the efferent pathways to the heart, without having to address off-target effects from the afferent pathways. A bipolar platinum stimulating electrode was placed on the right vagus nerve, distal to the crushed portion to create a perturbation to test the controller; the CSINE was placed distal to the stimulation electrode, as described previously [36]. A diagram of this setup can be seen in figure 1.

### 2.2. Pig surgical preparation

Pig experiments were performed under the approval of the University of California—Los Angeles (UCLA) IACUC under protocol 2016-084. UCLA is also an AAALAC-accredited institution conforming to all applicable federal, state, and local laws and regulations, in addition to institutional policies. Our team was trained and certified by qualified UCLA staff and adheres to Public Health Service policy and the Guide for the Care and Use of Laboratory Animals [47] when working on UCLA IACUC-approved protocols. A total of 5 Yorkshire pigs were used, weighing 50–60 kg. Pigs were sedated with midazolam (1 mg kg^−1^, intramuscular) and ketamine (10 mg kg^−1^, intramuscular), were intubated, and were then ventilated at 10–12 breaths per minute (300–450 ml tidal volume). Pigs were maintained under isoflurane (1%–4%) during the surgical procedures were completed and then were transitioned to alpha-chloralose (25–50 mg kg^−1^ hr^−1^, intravenous) for the nerve block trials. Alpha-chloralose was used as it has does not depress cardiac autonomic reflexes as compared to inhaled anesthetics. The cervical portion of the vagus nerve was exposed bilaterally but was left intact on both sides. The vagus nerve was minimally handled with no transection or crush injury, as to replicate a clinical environment where the vagus nerve was managed surgically with no alterations. On the right vagus, the bipolar stimulation cuff was placed at the proximal end of the exposure, and a CSINE was placed distally. A diagram of the setup can be seen in figure 1.

### 2.3. Differences in preparations between animal models

In the initial rat experiments, isoflurane was used, despite its known effects on the cardiovascular system [48] and axon dynamics [49, 50]. This was deemed acceptable, as the rat experiments were not designed to find final clinical parameters, but simply to test the treatment modality. The rat model also had the vagus nerves crushed (left side, proximal to stimulation and block) and cut (right side) to remove any afferent signals, produced due to the perturbation stimulation; these also removed the tonic efferent activity. This was done to isolate the system to get complete control over the vagus activity and followed the protocol we had done in the past to explore this relationship between block and HR [36]. Removing afferent and tonic activity on the vagus was important to isolate the effects produced by the block by preventing any confounding effects from reflexive activity.

To address these shortcomings of the rat model, we altered the protocol for the pig model to better reflect clinical conditions. Alpha-chloralose was used as the intra-experimental anesthetic, as it has been shown to better preserve cardiac function compared to isoflurane [51]. The vagus nerve on both sides was also left intact to better mimic a clinically-relevant scenario. While the differences between the two models make it hard to draw comparisons between the two models, these experiments demonstrate that closed-loop control was able to successfully control HR by modulating block of the vagus nerve. The changes made to the pig model made the data closer to a clinical application, and therefore easier to extrapolate to potential human use; this was deemed more valuable than retaining a limiting protocol to facilitate comparison between the two animal models.

### 2.4. Controller

The controller used in these experiments was a FLC, which translates discrete descriptions and observations of a system into a continuous input-output scheme. This was valuable in this case, as the cascade of interactions between the blocking of the vagus nerve to the effect on HR has many steps, each with a plethora of time constants and delays that vary from subject-to-subject. Defining a continuous model of this system and its many steps in detail would be difficult, as the exact characteristics of the system depend on many factors, such as time of day, activity level, sedation, and general variances from animal-to-animal. A FLC capable of adapting and responding to these differences and changes in real-time was then deemed to be a suitable fit for these tests. A more traditional proportional-integral-derivative (PID) controller was tested as well but did not perform well in initial testing due to the lengthy and variable delays seen in this system, as well as intra-animal differences. It may be possible to do more per-animal tuning of a PID to achieve adequate control, but for these experiments, a single, consistent controller with minimal changes was desired for repeatability and robustness.

**Figure 2.**
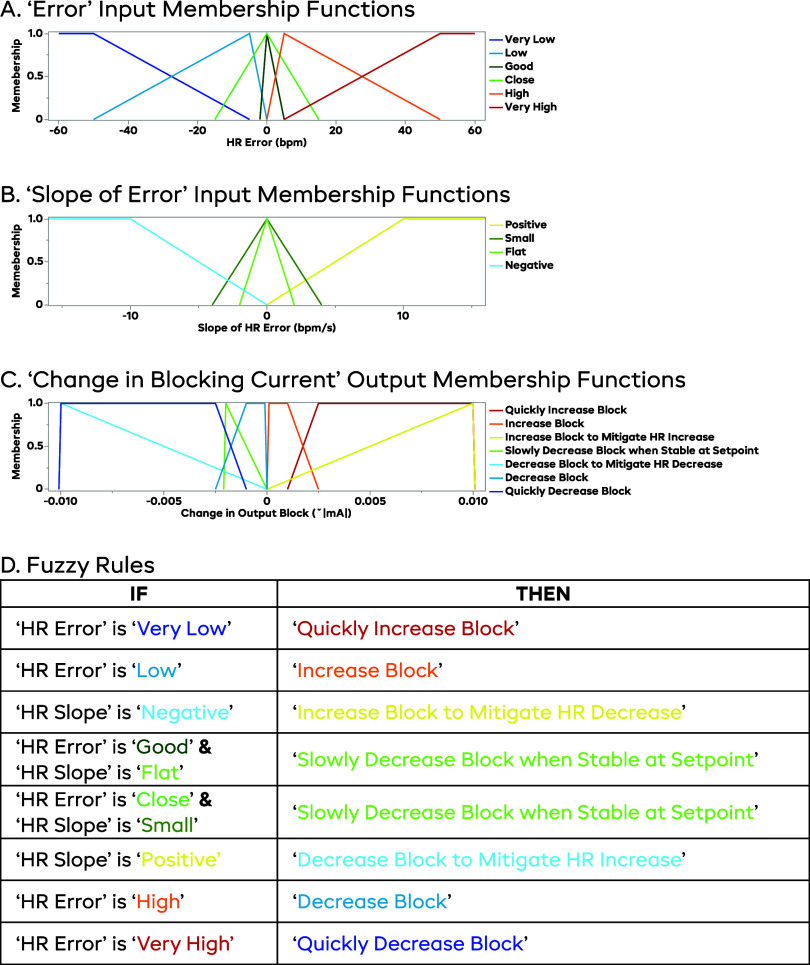
Illustration of the fuzzy logic controller. Subplot (A) shows the input membership functions for the error in heart rate (i.e. actual value—setpoint, bpm). Subplot (B) shows the input membership functions for the slope of the HR error (bpm/s). Subplot (C) shows the output membership functions for the controller (change in mA, where a positive value increases the amount of cathodic blocking current). Subplot (D) shows the list of controller rules, relating the input and output functions. The controller uses a ‘center of sums’ defuzzification method. The main theme of this controller is to increase the block if the HR is above the setpoint, decrease the block if the HR is below the setpoint, and to slow the changes to the block if the HR is trending in the correct direction. A unique feature of this controller is that the blocking current is lowered when the HR is near the setpoint, to counteract the property of DC block that causes it to increase in efficacy over time.

The controller designed for these experiments had inputs of instantaneous HR error as compared to the SP (ΔHR, measured HR—SP) and the slope of HR error (ΔHR*_n_*—ΔHR*_n_*_-1_). These inputs tell us how far away the measured HR is from the target HR, and if we are trending towards the SP or not. Heart rate was calculated by inputting three-lead ECG (rats) or left ventricular pressure measurements (pig) into a USB-6363 data acquisition device and LabVIEW (v.2022, NI, Austin, TX, US) software was used to calculate HR and implement the FLC. Using these inputs, the FLC decides what changes to the block level need to be made in real-time, allowing for closed-loop control of the system. The FLC-determined blocking current was output through a Model 2450 Source-Meter (Keithley Instruments, Cleveland, OH, US). The parameter space for the FLC can be seen in figure 2. For example, if HR is below the SP, and is dropping, the block level would increase quickly to reduce the parasympathetic inputs. As the HR begins to trend towards the SP (but still below it), the rate at which the block amplitude increases is reduced; the rate of increase is further reduced as the HR gets closer to the SP. Once the HR reaches the SP, the FLC will begin to slowly lower the block amplitude, to account for the *induction effect* increasing the amount of block seen over time. By slowly reducing the block amplitude, the FLC can effectively counter this effect and keep the HR at the desired SP.

The output of the FLC is a *change* in amplitude of the DC blocking waveform. This change was then multiplied by a gain, so that the controller speed could be adjusted easily with a single number. For the rat experiments, this gain was set to 2x, and pig trials were run at either 2x or 10x gain. Typically, with controllers, a higher gain will hypothetically allow you to reach the SP faster, as the controller will adapt faster; the tradeoff of a higher gain will be increased instability, as the controller may overshoot the SP, and need to reverse course to bring the HR back to the SP. This overshoot behavior, if bad enough, can lead to persistent oscillations in both the blocking amplitude and the HR, neither of which is clinically desirable. A lower gain will take longer to reach the HR SP but will be less prone to oscillations. A balance must be struck between the speed and the stability of the controller to ensure that the block behaves as desired and does not cause any additional cardiac instability.

### 2.5. Experimental aims

The main goal of these experiments was to demonstrate the efficacy of closed loop control as compared to static block with no feedback adjustment (open loop control) in reaching and maintaining a target HR SP in the face of a synthetic overactive vagal tone from the proximal stimulation electrode. In each set, trials consisting of a static 10% of the 60 s (BT_60_, the current it took to completely block the nerve in 60 s, defined below) and a static 50% BT_60_ were compared to trials utilizing a controller with the HR SP selected to be 50% of the change in HR in response to the VNS perturbation. As mentioned above, two different controller gains were used for some pig experiments, and one trial at each gain was included in each set.

The FLC system described in this study was designed to be both *relatively simple* and *robust across all animals*, with no per-animal tuning required. The FLC used was based primarily on past experiences and intuition about the nerve block dynamics, and minimal time was required to tune the system. These experiments were not designed to optimize the control schema for each individual animal. As such, we recognize that more complicated control paradigms (or even a differently tuned FLC) may achieve more precise results, in both individual examples and across all examples. For any given application, a balance between complexity vs functionality must be considered. This study aims simply to highlight the potential for closed-loop electrical nerve block for treatment of cardiovascular disease, and that this treatment paradigm did not need to be altered significantly across the species tested.

### 2.6. Experimental protocol

After the VNS (perturbation) and block (therapy) electrodes were placed on the vagus, stimulation on the VNS electrode was performed to determine the stimulation parameters needed to generate a measurable change in the HR. In the rat, 30 Hz, 50 *μ*s pulses were applied using current controlled stimulation (S88 stimulator with PSIU-6 isolation units, Grass Instruments, West Warwick, RI), and the amplitude of the stimulation was increased until the maximum effect on HR was achieved. In some cases, onset of severe bradycardia with a precipitous drop in HR (to HR sometimes below 100 bpm in the rat)was seen at a specific amplitude. When this occurred, the highest amplitude that did not incur this drastic change was used.

On the pig, 10 Hz, 1 ms pulses were delivered using the same equipment. In the rat, an acceptable change in HR was said to be greater than 25 bpm based on preliminary experiments and data analysis, so that the differences in the controller and the static values would not be overshadowed by the background variations in HR, especially due to respiratory sinus arrhythmia. In the pig, stimulation amplitude was increased until a 15% decrease in HR was seen following 30 s of stimulation; for the subsequent trials, 1.5 time this amplitude was used to ensure that a large enough change in HR was seen. The 15% level was chosen as a balance between a large enough response to measure, and to not be so large that the pig would become unstable. The rat subjects were able to handle larger swings in HR without adverse effects, so maximal response was used. If a suitable HR change were not achieved, the electrodes would be repositioned or replaced until a robust response was seen.

In these studies, VNS is not used as a treatment, but instead as a perturbation for the block electrode to correct. VNS increases activity on the vagus, while vagus nerve block decreases activity; while using both at different times may allow you to have more complete control over activity in the vagus, this study does not investigate this. We also do not propose to both stimulate and block at the same time as a treatment, as the same effect could be achieved by simply lowering the stimulation amplitude. By applying maximally effective (or the maximum comfortable dose) of VNS prior to the delivery of block, we simulate a worst-case scenario, where the block does not have any chance to mitigate the effects of an overactive vagus until the effect is at its strongest. If we were to allow the block to turn on prior to stimulation, it is likely that very little would happen in the case of static block, or that the HR would only be able to drop to a lesser extent in the controlled trials.

Once the HR perturbation was established, DC nerve block was delivered through the distal CSINE to the nerve to determine the ‘60 s (BT_60_)’. While applying proximal stimulation to lower the HR, increasing amplitudes of nerve block were tested until a level was found to bring the HR back to baseline in 60 s. If the block were able to return the HR to baseline faster than 60 s, the amplitude of the block was reduced so that the HR would not reach baseline until exactly 60 s. Similarly, if the HR did not return all the way to the baseline value in 60 s, the blocking amplitude was increased to promote a higher degree of block. The BT amplitude that achieved this was called BT_60_. Especially in pigs, the inherent delays of the cardiac system would often slow down changes in HR, meaning that it is impossible to measure quick changes in block efficacy. A 60 s period was chosen to assess the block, as this was long enough to be achieved in all subjects, as some would take up to this amount of time to recover when stimulation was turned off without block. This protocol was the same in rats and pigs, despite the rat HR being seen to change faster. An example of BT trials can be seen in figure 4(A).

For the static trials, the applied amplitude was a proportion of the measured BT current, BT_60_. For example, if BT_60_ was found to be −2 mA, a 10% BT_60_ trial would apply −0.2 mA, and a 50% BT_60_ trial would use an amplitude of −1 mA. This was done to normalize each experiment to the current it took to block for the specific experimental preparation. The controller output is also scaled in proportion to the BT, so all blocking currents were normalized to the same value. We chose 10% and 50% as the tested proportions of BT_60_ based on preliminary data which showed that the controller would most often settle between these two values. Only two values were chosen to limit the number of trials needed to be performed in a single data set; this served to increase the chances that enough trials could be performed in each animal, which in turn reduced the number of animals needed to complete the study. These values were far enough apart to also provide good illustrative examples of the induction effect (especially at 50% BT_60_) and that the induction effect has a lower limit (seen in most 10% BT_60_ trials). Finally, 50% BT_60_ was chosen to illustrate that halving the blocking current does not halve the block effect, as the relationship between the blocking current and effect is non-linear.

Data collection was performed using a Power1401 data acquisition device and Spike2 v.10 software (Cambridge Electronics Design, Cambridge, EN, UK). Data collection trials consisted of the following steps:

(1) Measuring a baseline HR
(2) Applying VNS to measure lowest HR (2 min in rats, 30 s in pigs)
(3) Applying static or controlled block and measuring response (10 min in both rats and pigs)
(4) Turn block off and measure recovery of HR to VNS levels (until HR recovers)
(5) Turn VNS off and let the HR recover to baseline.

**Figure 3.**
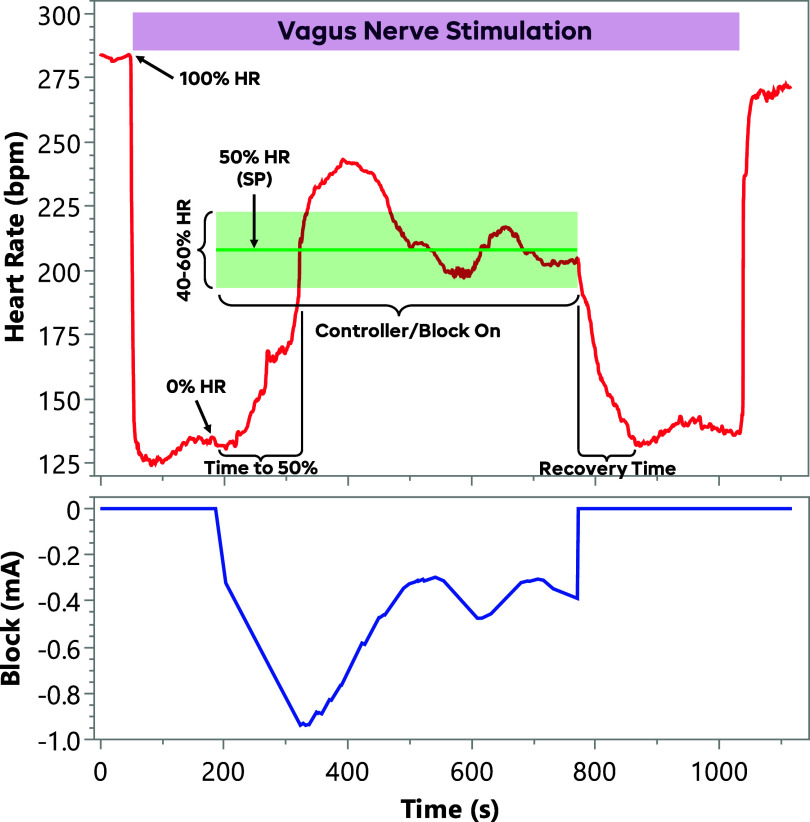
Example controller trial in a rat. The subplots (A) show the heart rate and block for a typical controller trial. The baseline heat rate for any therapy is applied is defined as the ‘100% HR’ for analysis. Vagus stimulation is then turned on, and the HR after 2 min is the ‘0% HR.’ After this, the static or controlled block is turned on (while leaving the VNS on) for 5 min. After the block was turned off, the vagus stimulation was left on to watch the HR recover to the 0% HR. The stimulation was left on for at least 2 min after the cessation of block. In the analysis, the time to 50% was the time from the time the block or controller started to the point when the HR crossed the ‘50% HR’ (halfway between the 0% and 100% HRs). The ‘time at 50%’ was defined as when the heart rate was within the light green box (from 40% to 60% HR). The ‘recovery time’ was the time from the cessation of block to the point when HR has returned to the 0% HR level. After determining recovery time, the VNS was stopped, and the HR recovered to the baseline HR. Some oscillation is seen in both the heart rate and the blocking current, indicating that the gain may have been too high in this system. The controller begins to settle at the setpoint by the end of the trial.

An example trial can be seen in figure 3(A). These trials were organized into sets containing a static 10% BT_60_ block trial, a static 50% BT_60_ trial, and an FLC-controlled block trial (gain = 10). Some sets of pig data included a FLC-controlled trial with a gain of 2. These trials were randomized within each set.

### 2.7. Data extraction

After collection, the data were analyzed using the Spike2 software. For all trials, the *baseline HR* was defined as the HR immediately before applying the initial VNS. The *VNS HR* was similarly defined as the HR with VNS applied right before the block was turned on (either the static level or starting the controller output). These values were used as the 100% and 0% HR, respectively, for setting the *50%* controller *SP* and for normalizing the HR across trials and experiments. The *time at 50% level* was defined as the total time when the block was on where the measured HR was within 10% of the normalized 50% HR (i.e. between 40% and 60% levels of normalized HR). This level was chosen to find a balance to be on the order of normal HR changes seen in every day, such as when standing which increases HR 10–20 bpm in humans [52], (∼10%–30%), but to not be majorly effected by small changes in HR, such as those due to respiratory sinus arrythmia [53], (∼3% [54]). The *recovery time* was defined similarly to previous publications [36] as the time to reach the VNS level from the moment the block was turned off. The *delivered charge* was also calculated by taking the integral of the amplitude during the blocking period. Illustrations of these metrics can be seen in figure 3. The *normalized HR traces* during the blocking periods were also compiled for comparing the average trajectories of the HR during each trial.

Statistical analyses were performed in JMP Pro version 18.1.0 (JMP Statistical Discovery, Cary, NC). Preliminary analysis showed that measurement data was not normally distributed; as a result, the nonparametric Wilcoxon test was used to compare category pairs throughout, with a statistical significance level of *α* = 0.005 [55]. Raw and analyzed data can be found on the Harvard Dataverse repository [56].

## 3. Results

### 3.1. Block threshold

**Figure 4.**
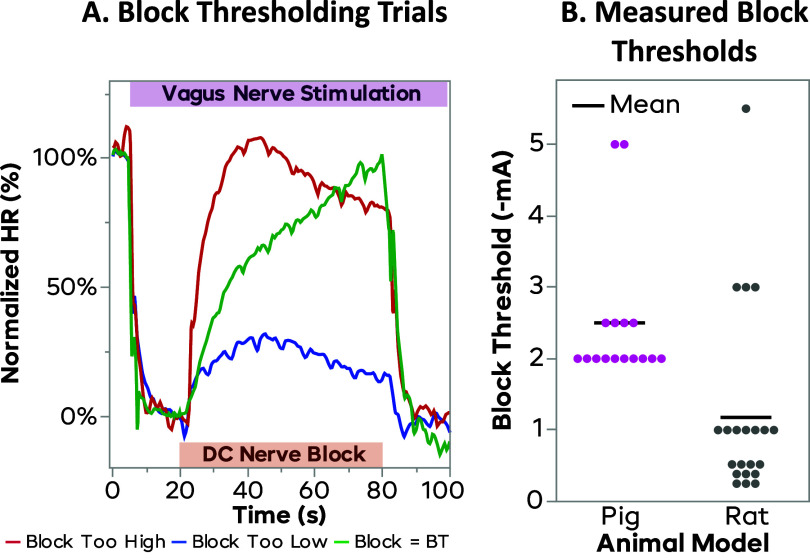
Block thresholds for each set for both rat and pig. *Subplot (A)* shows example trials during block thresholding. Vagus stimulation was applied to lower the HR to the 0% level, and the block is applied to raise the HR back to the baseline (100%) level in 60 s. The red plot shows the HR rising too quickly, the blue line does not reach 100%, and the green line reaches 100% in exactly 60 s, and is deemed the block threshold. *Subplot (B)* shows the block thresholds for both the rat and pig models. The black line indicates the mean block threshold. The rat block thresholds were slightly lower than the that of the pig (means 1.18 and 2.50 mA, respectively) but had a larger spread (standard deviations 1.35 and 1.00, respectively). These models were deemed to behave similarly enough for the controller to successfully port from the rat model to the pig, despite the differences in nerve size and electrode tips.

Figure 4(B) shows the distribution of BT for both the rats and pigs. The rats had a mean BT of −1.18 mA, with the smallest observed BT being −0.25 mA and the largest being −5.5 mA. The pig had a mean BT of −2.5 mA, with the smallest being −2 mA and the largest being −5 mA. This indicates that the rats typically have a lower BT but are also more variable when compared to the pigs. Due to the difference in nerve size, electrode designs were adapted for each animal model explaining some of the variability. A Wilcoxon test showed that these distributions are significantly different (*p* = 0.0007). Despite these *statistical* differences, the overlapping range lead us to conclude that there were not *practical* differences, and that controller and methodology were suitable to port from rat to pig.

### 3.2. Rat

#### 3.2.1. Averaged trials

**Figure 5.**
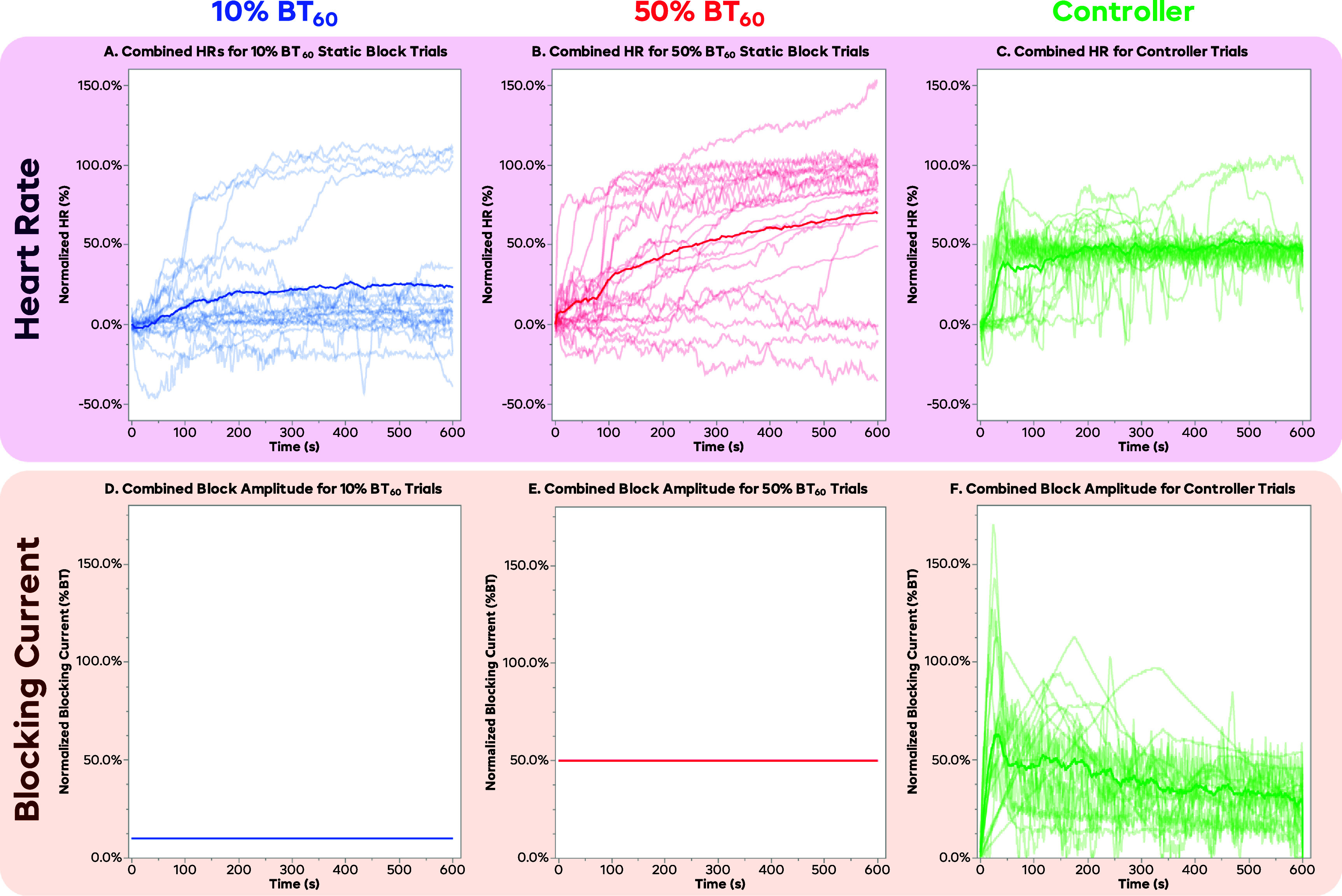
Combined results from the rat trials. Plots (A)–(C) show the normalized HR for the 10% BT_60_, 50% BT_60_, and controller trials. Each light trace shows an individual trial, while the darker trace shows the mean trace across all time points. The 10% BT_60_ trials show the least amount of block, with most being under 25%; however, a few trials had significant induction effects and were able to completely block back to the 100% baseline. The 50% BT_60_ trials show a wide array of responses, with significant amounts of trials achieving both little-to-no block and achieving complete block. The spread of these trials indicates very low predictability. The FLC controller trials show a leveling off of the HR at 50% for most trials, with a substantially smaller standard deviation. Several trials showed spontaneous changes in the heart rate throughout the trial; however, in most cases, the controller was able to adapt and bring the heart rate back to the setpoint during the duration of the block. Plots (D)–(F) show the combined normalized %BT for the 10% BT_60_, 50% BT_60_, the controller trials, respectively. The static trials seen in (D) and € are simply flat lines, as the same normalized amplitude was used for every trial. Plot F shows the combined normalized %BT for the controller trials, showing that the block typically spikes above 50% at the start of the trial, but continually decreases throughout the trial. The spread of the blocking currents is quite varied, showing the need for closed-loop control to achieve a constant setpoint.

Figure 5 shows an averaged result for all trials from each trial type: 10% BT_60_ static, 50% BT_60_ static, and the 50% SP FLC-controlled trials. The HR was first normalized so that the baseline HR just before initial VNS was 100%, and the resultant VNS HR just before block began was 0%. This is the same method used to define the 50% SP for the controller. The HR was sampled in each trial every 0.5 s, and the solid line represents the mean of all the trials at each time point. Figure 5(A) shows the results from the 10% BT_60_ static trials; the average block at the end was approximately 25%, with a standard deviation of ∼45%. Negative values of HR indicate that the HR fell further below the VNS level at the beginning of the trial. This was either from increased efficacy of the VNS over time, or from a natural HR drift during the span of the trial. In general, 10% BT_60_ was not enough to reach the desired 50% HR level. The 50% BT_60_ static results are shown in figure 5(B), showing a mean HR of ∼70% at the end, with a standard deviation again around 45%. This shows that while 50% BT_60_ was able to reach the 50% HR level, there was a lot of inter-trial variability, and that even within a trial there was not a high degree of control. It also took until ∼200 s for the mean HR to reach the desired 50 ±10% HR. Note that in the static trials, the maximum normalized HR recorded was roughly 100% (baseline); however, in some trials, the animal experienced some drift in the baseline HR, allowing the HR to go significantly above 100%. The 50% SP controlled trials in figure 5(C) showed an average HR of 45%, with a standard deviation of ∼15%, with the mean reaching the 50 ± 10% normalized HR in under 100 s. These show that not only was the controller more consistent at achieving a set value faster, but it was able to maintain the desired HR more precisely as well.

Figures 5(D)–(F) show the manually set amplitude of block for the static-block trials, and the amplitude set by the controller for the FLC trials as a proportion of BT_60_. The static trials predictably are just flat lines at their respective amplitudes (figure 5(D) at 10% BT_60_ and figure 5(E) at 50% BT_60_), as the same constant amplitude was used throughout each trial. Controller trials seen in figure 5(F) tended follow a similar shape of quickly ramping up to a peak of on average 63% BT_0_ about 30 s into the trial, then slowly decreasing amplitude throughout the trial, ending the 10 min of block at an average amplitude of 29% BT.

#### 3.2.2. Time at 50% HR

**Figure 6.**
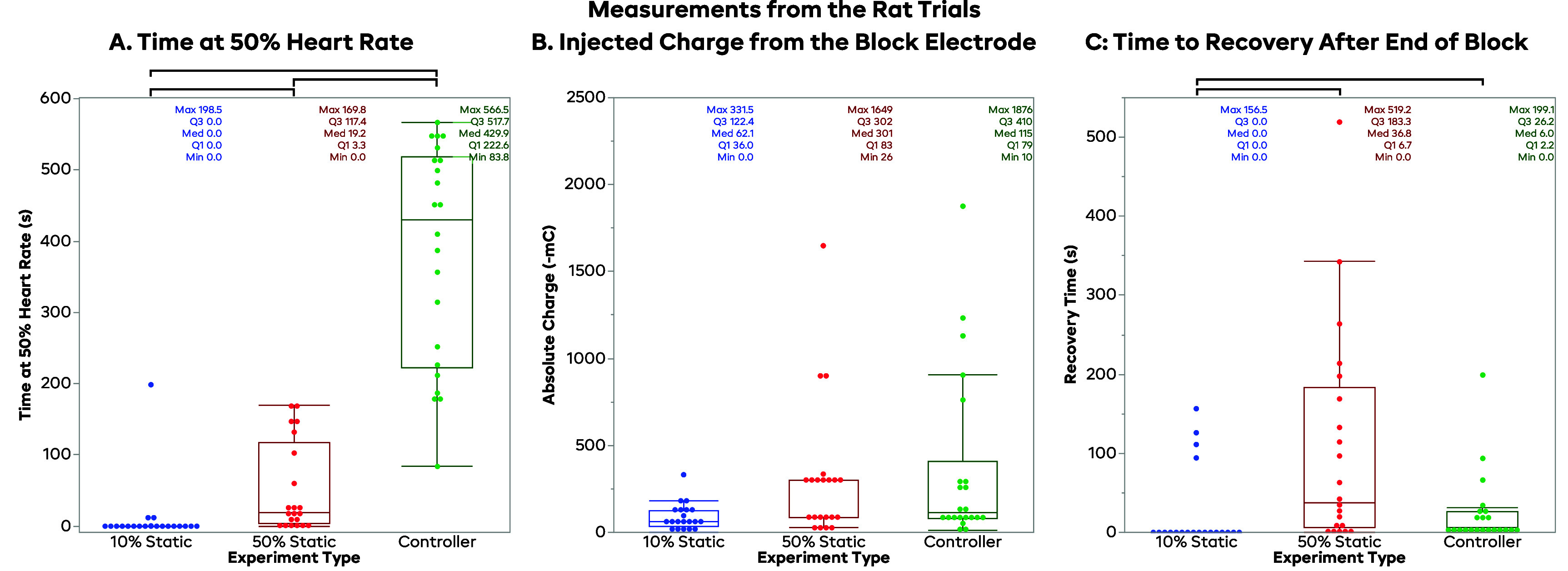
Output measurements for the rat. Black brackets indicate a statistically significant difference in the Wilcoxon test for that pair. *Subplot (A): time at 50% heart rate for the rat trials*. The plot above shows the time at 50% BT_60_ for each of the trial types, with the controlled trials spending significantly more time at the 50% HR (median 398.3 s) than the uncontrolled trials (10% median 0 s; 50% median 19.2 s). *Subplot (B): injected charge from the block electrode for the rat trials*. This plot shows distributions for the injected charge of the block electrode for each the trial types, with the 50% BT BT_60_ having the most on average, but with a larger range in the controller trials. The 10% static trials had a median of 62.2 mC, the 50% had a median of 301.0 mC, and the controller trials had a median of 115.0 mC. *Subplot (C): time to recovery after end of block for the rat trials*. This plot shows the time to recovery for each of the trial types, with the 50% BT_60_ having a considerably longer recovery time (median 36.8 s) compared to the 10% static (median 0.0 s) and the controlled trials (6.0 s).

Figure 6(A) shows the distribution of times that each trial type maintained the HR near 50% (between 40% and 60%). The 10% BT_60_ static trials had a median of 0 s at the 50% HR, mainly due to the fact that most of the trials did not block enough to reach 50%. The 50% BT_60_ static trials had a median of 19 s at 50% HR, with some trials not making it to the desired HR, and some going above it. This illustrates the low degree of predictability with a statically applied blocking current. The FLC-controlled trials had a median time of 398 s within 10% of our SP, much higher than either of the static block levels, demonstrating the effectiveness of the control scheme. Wilcoxon tests on each pair of trial types showed statistically significant differences in the distribution between all pairs (10% BT_60_–50% BT_60_, *p* = 0.0001; 10% BT_60_—FLC, *p* < 0.0001; 50% BT_60_—FLC, *p* < 0.0001). As a course measurement of controller efficacy, we defined a *successful* controller trial as being able to maintain the HR at the SP (between 40% and 60%) for half of the trial, or 300 s. Using these criteria, 15 of the 22 controller trials (68.2%) were considered to be successful.

#### 3.2.3. Charge delivered

Figure 6(B) shows the distribution of injected charge for each of the trials. The 10% BT_60_ and 50% BT_60_ trials were simply related to BT_60_ since the times and amplitudes were constant; the FLC trials had a higher potential for variance, since the amplitude was varied by the controller. The median charge delivered for the 10% BT_60_ trials was 62 mC, with a mean and standard deviation of 89 mC and 77 mC, respectively. The 50% BT_60_ trials had a median of 301 mC, with a mean and standard deviation of 309 mC and 394 mC, respectively. The FLC trials had a median of 115 mC of injected charge, and a mean of 365 mC and a standard deviation of 497 mC. These illustrate that for the rat, the injected charge of the FLC varied more than the differences caused by the variability of BT, and that the total injected charge of the FLC was not much different than the 50% BT_60_ trials, despite their different outcomes. While there may be some differences in injected charge in specific subjects, the CSINE was well within the charge capacity of the device throughout the duration of the experiment without a recharge. Wilcoxon tests on each pair of trial types showed no statistically significant differences in the distribution between any pairs (10% BT_60_–50% BT_60_, *p* = 0.0127; 10% BT_60_—FLC, *p* = 0.0098; 50% BT_60_—FLC, *p* = 0.7246).

#### 3.2.4. Recovery time

Figure 6(C) illustrates the time to recovery back to the VNS HR after the block was turned off. The 10 BT_60_ trials have a median of 0 s for the rat and a mean of 24 s, due to many of the trials not blocking at all, and many only blocked slightly and thus recovered quickly. The median recovery time for the 50% BT_60_ trials was 37 s, or roughly 10% of the applied 6 min of block, with the mean being 107 s, or roughly 30% of the applied block time. The FLC trials had a median recovery time of 6 s and mean of 25 s, considerably smaller than the 50% BT_60_ trials, despite a more consistent degree of block. The 50% BT_60_ trials illustrate the tendency for static values to cause extended carryover once complete block is achieved. Wilcoxon tests on each pair of trial types showed statistically significant differences in the distribution between the 10% BT_60_ & 50% BT_60_ (*p* = 0.0006) and the 10% BT_60_ & FLC (*p* = 0.0017) distributions, but not between the 50% BT_60_ & FLC distributions (*p* = 0.0277). A Pearson correlation test did not indicate that charge delivery was correlated to recovery time in the controller trials (*r* = −0.03209, *p* = 0.8873).

### 3.3. Pig

#### 3.3.1. Averaged trials

**Figure 7.**
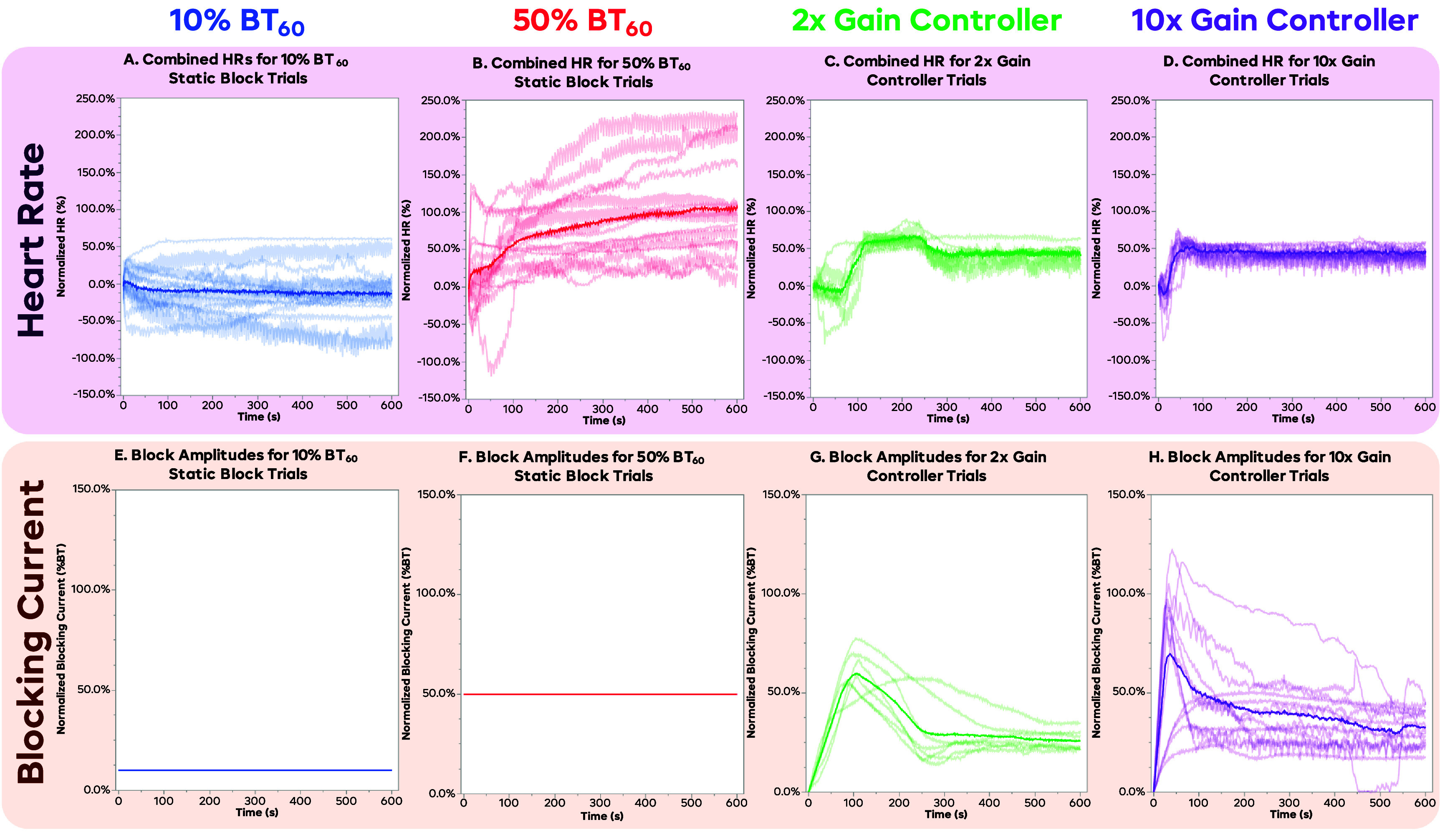
Results from the pig trials. Plots (A)–(D) show the mean normalized HR for the 10% BT_60_, 50% BT_60_, the 2x gain controller, and the 10x gain controller trials, respectively, with the lighter lines representing individual trials, and the darker line representing the mean value at each time point. The 10% BT_60_ shows the least amount of change with large spread. The 50% BT_60_ plot show that the average trial goes up to the 100% HR in about 5 min, and begins to level off from there, albeit with a large amount of trial-to-trial variance. The 2x gain controller trial shows an increase to about 75% HR, and then a correction back to the 50% setpoint, with substantially smaller trial-to-trial variance. The 10x gain controller shows a slightly better-controlled curve, reaching and settling at the 50% setpoint quickly. Plots (E)–(H) show the combined normalized %BT for the 10% BT_60_, 50% BT_60_, the 2x gain controller, and the 10x gain controller trials, respectively. The static trials seen in (E) and (F) are simply flat lines, as the same normalized amplitude was used for every trial. The 2x gain controller (G) increases the block to a peak neat 60% and then reduces to about 30% by the end. The 10x gain controller (H) peaks slightly higher near 70%, and settles slightly higher as well, at about 35%.

Figure 7 shows the averaged trials for the porcine experiments, similar to figure 5 for the rat data. Figure 7(A) displays the averaged data for the 10% BT_60_ trials, which again show a low block at the end of the trial, with the HR decreasing on average to roughly 13%, with a standard deviation of ∼37%. The 50% BT_60_ trials behaved similarly to the rat as well, but with a slightly more effective average HR at the end of around 105% with a standard deviation of 68%, as seen in figure 7(B). Since the vagus nerves were left intact for the pig experiments, there will be some tonic vagal activity that can be blocked to raise HR above baseline. The 50% BT_60_ trials took an average of 70 s to reach the 50 ± 10% goal value. The FLC trials with a gain of 2 (figure 7(C)) had an ending HR of 45%, with a standard deviation of 8%, and took approximately 100 s to reach the 50 ± 10% goal value. The FLC trials with a gain of 10 (figure 7(D)) had an ending HR of roughly 44% with a standard deviation of 12% and took roughly 40 s to reach the goal value. Due to the use of sub-maximal stimulation, and only 30 s of stimulation beforehand, a HR below 0% was more common in unblocked nerves, as the VNS would continue to lower the HR after the block was turned on. Similarly, leaving the vagus nerves intact resulted in a higher rate of HR above the 100% normalized level. We believe this is in part due to the blocking of tonic vagal activity that was present in the pigs but was not present in the cut and crushed rat nerves. Baseline drift also played a role in these, similar to the rats.

Figures 7(E) and (F) show the averaged current amplitudes for the static 10% BT_60_ and 50% BT_60_ trials, respectively. As in figure 5, these are just flat lines, as the same amplitude is used throughout each trial. Figures 7(G) and (H) shows the averaged block amplitude for each of the gains of the controller, as a percentage of BT_0_. The 2x gain controller block level (figure 7(G)) increased for about 105 s to a peak of 60% BT_0_, then falling quickly to 29% BT_0_ at 270 s, and then leveling out but slowly dropping to 26% BT_0_ by 600 s. The 10x gain controller block level (figure 7(HS)) followed a shape more similar to the rat trials, peaking on average at 70% BT_0_ at 35 s into the trial before more steadily decreasing to 32% BT_0_ at the end of the trial.

#### 3.3.2. Time at 50% HR

**Figure 8.**
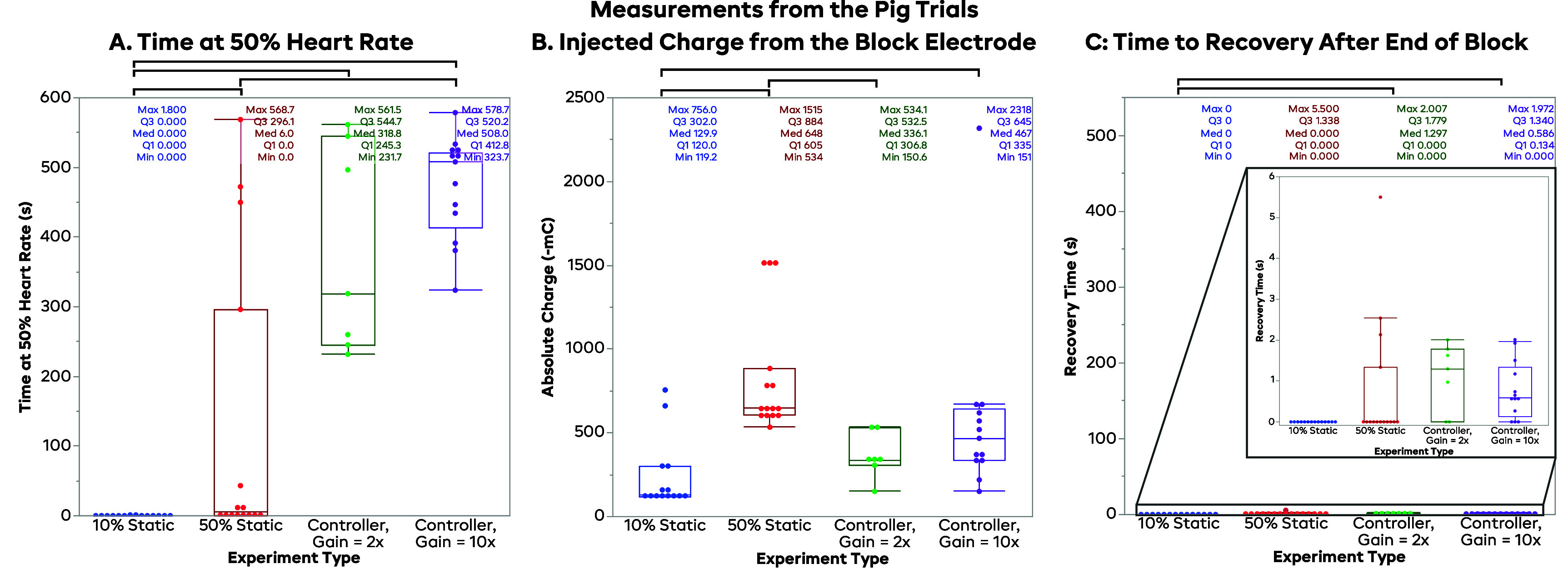
Output measurements for the pig. Black brackets indicate a statistically significant difference in the Wilcoxon test for that pair. *Subplot (A): time at 50% heart rate for the pig trials*. The plot above shows the time at 50% HR for each of the trial types, with both controllers performing better on average than either of the static trials. The 10x gain controller (median 508.0 s) performed slightly better than the 2x gain trials (median 318.8 s), likely due to their ability to rise faster. While the average for the 50% BT_60_ static block trials is quite low (median 6.0 s), there were a few trials that did appear to stabilize around 50% HR for an extended period. None of the 10% BT trials spent any significant period near our target zone (median 0.0 s). *Subplot (B): injected charge from the block electrode for the pig trials*. This plot shows distributions for the injected charge of the block electrode for each the trial types, with the 50% BT_60_ static block (median 648.0 mC) having the most on average, followed by the controllers (10x median 467.0 mC; 2x median 226.1 mC), with the 10% BT_60_ static block coming in with the lowest charge (median 129.9 mC). *Subplot (C: time to recovery after end of block for the pig trials*. This plot shows the time to recovery for each of the trial types. The inset shows a zoomed-in *y* axis so that the data can be seen (original axes same as figure 8 for the rat). In the pig, no trials had particularly long recovery times, and even the worst point (5.5 s) would not be clinically significant. The median for both static trials was 0.0 mC, the median for the 2x controller was 1.3 s, and the median for the 10x controller was 0.6 s.

Figure 8(A) shows the distribution of the times at our 50 ± 10% target HR. The median time that the 10% BT_60_ trials spent in the target zone was 0 s. In fact, only 2 trials hit the target region at all for the 10% BT_60_ trials. The 50% BT_60_ trials faired a bit better, with a median of 6 s at the target HR; however, unlike the 10% trials which did not reach the target, these trials largely reached the target and quickly went above it, as seen in the averaged data in figure 7(B) The FLC trials with a gain of 2 (FLC 2x) spent a median of 319 s at the target HR, while the gain of 10 (FLC 10x) trials spent a median of 508 s at the target, nearly 85% of the 6 min trial. Wilcoxon tests on each pair of trial types showed statistically significant differences between the 10% BT_60_ & FLC 2x distributions (*p* < 0.0001), between the 10% BT_60_ & FLC 10x distributions (*p* < 0.0001), between the 50% BT_60_ & FLC 10x distributions (*p* = 0.0005), and between the 10% BT_60_ & 50% BT_60_ distributions (*p* = 0.0027). The other pairs were not seen to be significantly different (50% BT_60_—FLC 2x, *p* = 0.0159; FLC 2x—FLC 10x, *p* = 0.2346). Similar to the rat, a controller trial was defined as a *success* if the HR was maintained at the SP (between 40% and 60%) for half of the trial, or 300 s. Using these criteria, 4 of the 7 FLC 2x trials (57.1%) succeeded, while 13 of 13 FLC 10x trials (100%) met these criteria.

#### 3.3.3. Charge delivered

Figure 8(B) shows the injected charge of each of the trial types for the pig experiments. Again, the static 10% and 50% BT_60_ trials have their charge directly tied to the BT, while the FLC trials have a higher chance of variability. The 10% BT_60_ trials had a median charge of 130 mC, with a mean and standard deviation of 237 mC and 210 mC, respectively. The 50% BT_60_ trials had a higher injected charge with a median of 648 mC, and a mean and standard deviation of 835 mC and 363 mC, respectively. The FLC trials both injected less charge on average than the 50% BT_60_ trials. The median injected charge for the FLC trials with a gain of 2 was 336 mC, with a mean and standard deviation of 363 mC and 134 mC, respectively; likewise, the median for the 10x gain trials was 467 mC, with a mean of 584 mC and a standard deviation of 547 mC. The higher gain led to an increase in overshoot in the amount of block delivered, contributing to the increase in charge. Wilcoxon tests on each pair of trial types showed statistically significant differences between the 10% BT_60_ & FLC 10x distributions (*p* = 0.0024), between the 50% BT_60_ & FLC 2x distributions (*p* = 0.0003), and between the 10% BT_60_ & 50% BT_60_ distributions (*p* = 0.0002). The other pairs were not seen to be significantly different (10% BT_60_—FLC 2x, *p* = 0.0229; 50% BT_60_—FLC 10x, *p* = 0.0075; FLC 2x—FLC 10x, *p* = 0.2048).

#### 3.3.4. Recovery time

Unlike in the rat model, the pig experiments showed almost no delay in recovery to VNS HR after block was turned off, as seen in figure 8(C). The 10% BT_60_ and 50% BT_60_ trials both had a median of 0 s, and the 2x and 10x gain FLC trials had medians of approximately 1 s each. The maximum recovery among all trials was under 6 s, a negligible amount. Wilcoxon tests on each pair of trial types showed statistically significant differences between the 10% BT_60_ & FLC 2x distributions (*p* = 0.0006) and between the 10% BT_60_ & FLC 10x distributions (*p* < 0.0001). No other pairs were seen to be significantly different (10% BT_60_–50% BT_60_, *p* = 0.0453; 50% BT_60_—FLC 2x, *p* = 0.2057; 50% BT_60_—FLC 10x, *p* = 0.1209; FLC 2x—FLC 10x, *p* = 0.3798). However, as stated above, while there may be statistical differences, these distributions are not significant in practice. Pearson correlation tests did not indicate that charge delivery was correlated to recovery time in the 2x gain controller trials (*r* = − 0.229719, *p* = 0.5842) or in the 10x gain controller trials (*r* = −0.23464, *p* = 0.4403).

## 4. Discussion

### 4.1. Averaged trials and time at 50% HR

The averaged trials show that the FLC-controlled block was more consistent at reaching a set value than the static trials. Even beyond the time maintained near the target 50% HR, the inter-trial variability is reduced with active control, as would be expected. The ability to quickly ramp up to a higher current to reach the target SP and then back off to maintain the desired HR also allows for the time it takes to reach the desired level to be reduced; in the rat, these properties increased overall delivered charge, but in the pig, the charge was actually reduced when compared to the 50% BT_60_ trials. This could be due to 50% BT_60_ resulting in more effective block in the pig model than in the rat, as illustrated by the average ending values of the averaged trials in figures 5(B) and 9(B).

When comparing the success rate of the controlled trials (40%–60% for 300 of 600s), the rat experiment showed promising results. Even including the time it took to initially reach the SP from the starting 0% HR, most controllers were able to maintain the SP for over half the duration of the experiment. In most trials, both block amplitude and HR seemingly converged towards a steady-state value (as seen in figures 5(C) and (F)), so we would expect this success rate as the duration of control is increased. For the experiments in the pig, a slightly worse success rate was seen in the slower (gain of 2) controller (likely due to the slow initial time to SP, and a consistent overshoot), but still over half the trials were deemed successful. The faster, gain of 10 controller achieved a perfect success rate, a very encouraging result. As seen in the rat experiments, both controllers seem to converge towards steady-state buy the end of the trial, indicating that longer trials would increase the success rate.

The need to reduce the amplitude of the blocking current to maintain the same HR is related to the induction effect that has been explored previously [36, 57]. In short, the induction effect describes the property where a DC block at a constant amplitude will become more effective over time. To counteract this and remain at a stable level of block the amplitude must then be lowered. While the mechanisms are not fully understood, it has been hypothesized that the induction effect (as well as the recovery effect) is in part caused by accumulation of K^+^ ions in the submyelin and perimyelin space [43, 46]. When an axon is depolarized by the blocking current, the Na^+^ inactivation channels close, but the K^+^ channels remain open and are constantly trying to bring the membrane back to resting potential. The constant leaking of K^+^ is hypothesized to alter the local ion concentrations around the membrane and eventually lead to block. These same concentration changes are also theorized to be responsible for the delayed recovery after block, as diffusion and the sodium-potassium pump need to slowly bring the ions back into balance. Others have said that longer pulses may elicit unique effects on ion channels compared to shorter pulses [35], which may also contribute to these effects.

The controller also showed a robust and repeatable response, as shown by the consistently lower standard deviation of the averaged trials when compared to the static block trials. Especially seen in figure 7(d) for the FLC 10x gain trials, even the amount of overshoot seen across trials was consistent. No trial was seen where the controller delivered an unsafe level of block; the controller did have a hard limit set to twice the BT, but the FLC never reached this value throughout these experiments.

### 4.2. Rat vs pig models

A major goal of these experiments was to investigate the translation of a controller system developed in rats to a larger animal model, in this case a pig model. In these experiments, a slight adjustment was needed to account for the differences in HR between the animals, but no other major revisions to the control scheme were needed. The data show that the controller was equally as effective in both the pig and rat models, with the 10x gain FLC in the pig being the most successful. The ease of translation from the rat to the pig model suggests that the controller design should be able to readily adapt to human use.

For both the rat and pig models, the approximate shape of the blocking current was similar: the block would quickly ramp up to a higher level of block until the HR begins moving towards the SP, at which point it would begin decreasing until the end of the trial. Both models (and both gains for the pig) have similar mean peaks and Both models (and both gains for the pig) have similar mean peaks and settling values; however, the rat at the 10x gain pig controllers appear to have more variability in the applied block at the beginning of the trial. In all cases, the variability of the output blocking current reduces, as the blocking level appears to converge near 30%. Differences in the convergence level could be due to slight inaccuracies or shifts in the BT or simply be due to animal-to-animal differences. However, the two animal models show remarkably similar trajectories for block, despite the significant difference in animal and nerve size.

### 4.3. Charge delivered

The average charge delivered showed that the controller delivered a lowered median charge than the 50% BT_60_ trials in both the rat and the pig. The mean current was higher for the rat FLC trials than the rat 50% BT_60_ trials; in the pig, the mean was lower for both types of FLC trials compared to the 50% BT_60_ trials. Both the mean and median of the FLC trials were higher than the 10% BT_60_ trials. Since injected charge is directly proportional to the average current, we can make the same observations about the blocking current needed in the FLC trials compared to the static trials.

### 4.4. Recovery time

In the rat, the recovery times of the FLC trials were again between the times for the 10% and 50% BT_60_ trials. This is an expected result given the injection of charge differences. However, the spread of the FLC trials’ recovery times was reduced when compared to the 50% BT_60_ trials. This is due to the ‘front-loading’ of the charge, where blocking currents are larger at the beginning of the controller trials but are lower towards the end. In a clinical application, a predictable recovery time would be desirable. Depending on the application, a longer or shorter recovery time may be desired, but regardless, a more predictable recovery is always beneficial. In the pig, all recovery times were short, with the longest time to complete recovery being 6 s. In the context of a change in cardiac output, this is virtually instantaneous, as there are intrinsic delays and biological limitations for how fast the HR can change. The difference in recovery times may be a result of the differences in the surgical preparation between the two animal setups. Since the left vagus and right afferent were left intact in the pig, the system effects may result in a faster recovery time. In the rat surgical preparation, the elimination of afferent and left side effects exposes the direct effect of the DC nerve block and its temporal properties. Neither the rat nor the pig models showed a significant correlation between charge injected and time to recover in the controller trials. This appears to go against our previous work blocking the rat vagus [36], although these experiments were not designed to test the effect of charge injection on recovery, and many of the controller trials injected a similar amount of total charge.

### 4.5. Effect of gain

In the pig, comparing the two gains for the FLC controller, we see both having success in achieving the target HR. This demonstrates the robustness of the controller design with respect to the gain, such that an exact gain is not clinically necessary. With controller design in general, it is imperative that one balances the speed of the response against the potential for oscillations, or ringing. In the case of this controller, the gain is the main factor influencing this balance. While most trials were seen to successfully reach the SP with minimal overshoot and ringing, oscillations were still seen in some trials (see the example in figure 3). In a clinical setting, where long-term stability is more desired than having a quick response, an overdamped controller with a lower gain may be desirable.

## 5. Conclusion

While vagus nerve stimulation has shown recent promise in treating diseases that benefit from an increase in vagal activity, electrical nerve block offers a treatment to lower vagus activity. This could help relieve chronic or intermittent bradycardia cause by heart disease or help treat acute conditions such as vasovagal syncope; the latter having few treatment modalities beyond the modest improvements from lifestyle changes or drugs with adverse effects [58]. Current surgical options for these diseases include nerve ablations, which are permanent, or implantable cardioverter-defibrillators, which only work after an arrythmia is detected. Since nerve block is reversable, it can provide the efficacy of an ablation when needed but allow for normal nerve conduction under normal circumstances.

Earlier studies using electrical nerve block typically use a static amplitude to stop conduction of the nerve fibers. While a static amplitude is sufficient for achieving complete block of a nerve, we have shown that static sub-block-threshold amplitudes vary in the overall magnitude of effect on the target organ (in this case, the heart), as well as change over time within an individual block application. Our closed-loop FLC was able to titrate the blocking current to achieve our desired SP quickly and maintain it near the SP better than static blocking levels. *Thus, we suggest that in applications where precise titration of DC nerve block is desired, closed-loop feedback is a requirement to achieve satisfactory results; static blocking amplitudes alone are not sufficient to address the temporal changes in block level.*

Previous work with DC block on the vagus showed that static blocks are susceptible to delayed induction to and recovery from block. Allowing the controller to ramp up quickly, and then quickly lower the amplitude increased the speed of the induction block. There may be some room for improving this further by starting the block not at 0 mA, but at some higher value. The controller was also effective in minimizing the current and charge needed to maintain a specific HR, which reduced the recovery time after block finished as well. The lower injected charge is also useful for an implanted system, as this would increase the battery life of the device, allowing it to remain on longer before a recharge is needed.

The FLC here was an uncomplicated design and was not tuned beyond preliminary testing. Despite this, the controller was able to successfully control the HR in both the pig and rat models, with no calibration or setup other than a rough estimate of the rat and pig HR. The success of this transition between animal models bodes well for the portability to human use. Future applications could utilize some subject-specific calibration of the controller to improve efficacy by, for instance, normalizing the controller outputs to the BT for each animal; however, the simplicity of a controller that does not require calibration could prove to be a more useful tool in some situations than a more accurate one that requires additional setup. Clinically, we could use historical data to estimate a calibration curve, which would allow for easy setup with the long-term advantages of a patient-specific calibration.

A key feature of the FLC that we anticipate will be helpful is its ability to quickly add different inputs and outputs to the system. This work focused on HR and vagus block, but other inputs (e.g. BP, EKG analysis, activation-recovery intervals, myocardial neurotransmitter levels) and outputs (vagus stimulation, sympathetic chain stimulation and block, drug delivery) may allow for more robust and precise control and allow control of the system to treat a wider range of cardiac disease.

## Data Availability

The data that support the findings of this study are openly available at the following URL/DOI: https://doi.org/10.7910/DVN/JVINZU.
